# Prolonged Inanition

**Published:** 1849-10

**Authors:** 


### PROLONGED INANITION.

Dr. Samuel H. London, of West Point, Tennessee, communicates to us the following case of prolonged inanition, in a hog, which, although not quite so remarkable as the instance often quoted of the pig buried under the chalk cliff in England, is extraordinary enough to entitle it to a place among the rare cases in physiology. Dr. L. says:

"A hog weighing about 190 pounds, the property of Mr. James Kelly of this neighborhood, by accident got wedged in between two logs and in that situation remained confined, without food or drink, for *ninety-six days*. When released the weight of the animal was reduced to *thirty* pounds, or more than five-sixths. The accident occurred during the past winter. At this time (June 10th) the hog is alive and as thrifty as any other on the farm."

